# Diverticular Perforation Secondary to a Chicken Bone: Food for Thought

**DOI:** 10.7759/cureus.4273

**Published:** 2019-03-19

**Authors:** Jacques G Eastment, Nick Butler, Kellee Slater

**Affiliations:** 1 Surgery, University of Queensland, Brisbane, AUS

**Keywords:** diverticular disease, diverticular abscess, colonic perforation, foreign body ingestion

## Abstract

A 56-year-old man presented to the emergency department with a one-day history of lower abdominal pain and fever. Clinical examination revealed generalized peritonitis. A computed tomography (CT) scan identified a linear hyperdensity straddling the site of a perforated sigmoid diverticulum. The patient proceeded to emergency laparotomy, which confirmed feculent peritonitis secondary to chicken bone perforation through the sigmoid colon diverticulum. After removal of the bone, Hartmann’s procedure was performed, and the patient subsequently made an excellent recovery.

## Introduction

Diverticulosis is a common gastrointestinal condition that can be complicated by inflammation or bleeding. Acute diverticulitis is thought to result from perforation of the colon secondary to erosion of the diverticula walls by increased intraluminal pressure [1]. Micro-perforations can cause pain that is often self-resolving, and these presentations are usually managed without intervention. Macro-perforations are typically associated with more complicated disease and can cause pelvic abscesses, fecal contamination, and peritonitis. One study has shown that in populations with endoscopically proven diverticulosis, approximately 4% of patients will develop diverticulitis [2]. In rare instances, diverticulitis can be caused by the ingestion of foreign bodies such as toothpicks or animal bones [3].

We present a case of feculent peritonitis secondary to chicken bone perforation at the site of a sigmoid diverticulum. The acute presentation was successfully managed with exploratory laparotomy and removal of the bone followed by resection of the perforated sigmoid colon and Hartmann's procedure.

## Case presentation

A 56-year-old male with no medical conditions presented to a hospital emergency department with a one-day history of increasingly severe and constant abdominal pain. The patient described associated fecal urgency with small, frequent bowel movements, subjective fevers, and rigors. He also reported earlier dysuria and anuria for several hours.

On examination, the patient was obese, hemodynamically stable, and afebrile. Initial abdominal palpation elicited tenderness in the right iliac fossa and suprapubic area. Blood tests demonstrated a raised white cell count (11.8x10^9/L) with neutrophilia (90%) but identified no anemia or biochemical evidence of end-organ dysfunction. 

Given the patient’s inability to pass urine, the treating emergency physician made a provisional diagnosis of acute urinary retention. Urinary catheter insertion drained only 100 mL of urine without symptom relief. Serial abdominal examination in the emergency department elicited increasing tenderness and the new development of lower abdominal peritonitis. The patient was transferred to a tertiary facility.

**Figure 1:**
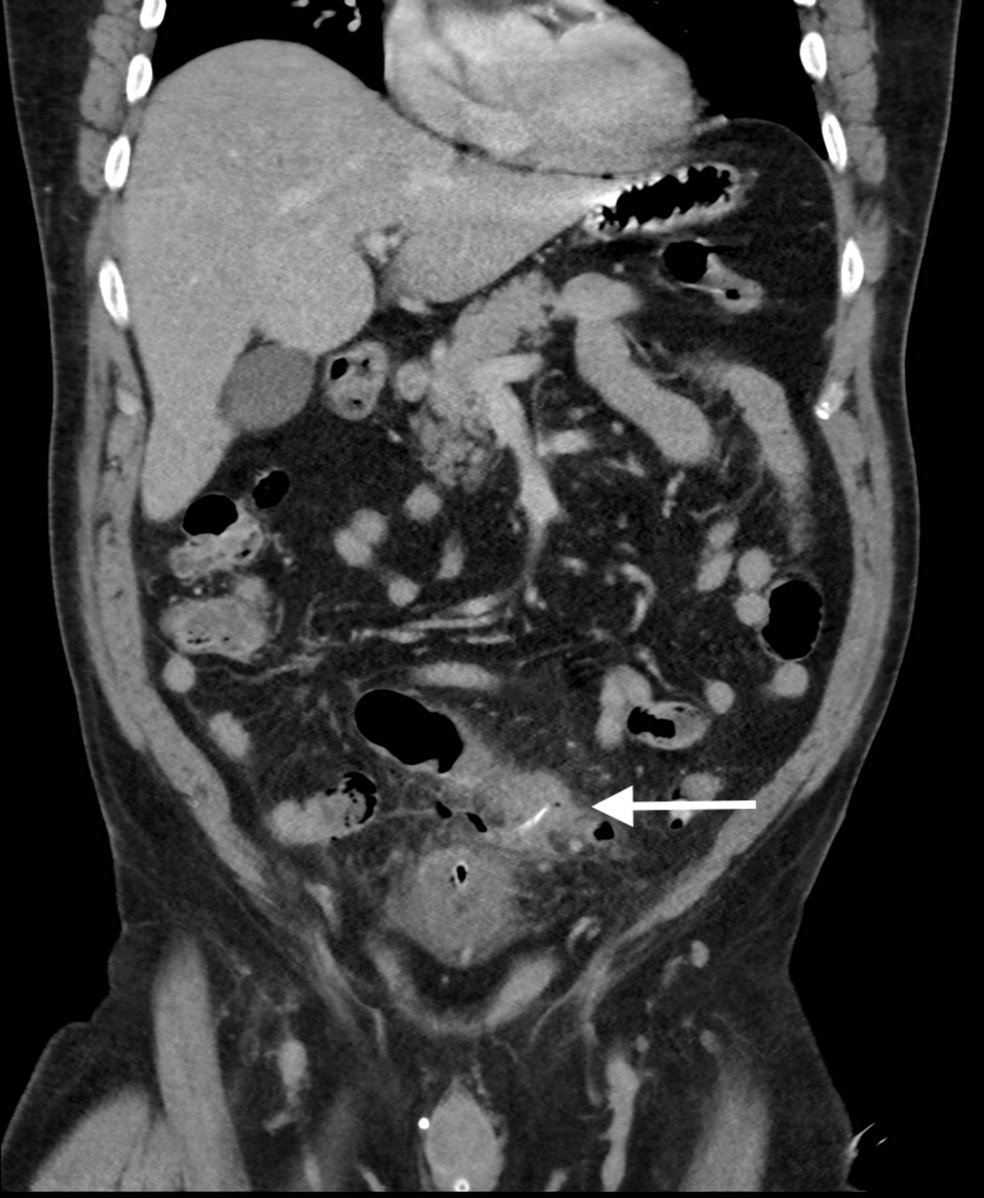
Coronal view of an abdomino-pelvic computed tomography scan. Linear hyperdensity is visible within the inflamed sigmoid colon (white arrow).

A contrast-enhanced computed tomography (CECT) scan of the abdomen was performed in the portal venous phase (Figure 1), revealing mucosal thickening and severe inflammatory changes within the sigmoid colon. A 19 mm linear hyperdense focus was visible at the center of the most inflamed region of the sigmoid colon. This appeared to traverse the diverticular wall and was associated with a locule of free gas. These findings were consistent with foreign body perforation of a sigmoid diverticulum.

On further history, the patient recalled eating chicken for lunch but could not recollect swallowing a bone. Upon arrival at our tertiary facility, the patient had become tachycardic, tachypnoeic, and febrile, and showed signs of generalized peritonitis. The patient was taken for urgent laparotomy. Findings were of a perforation of a sigmoid colon diverticulum by a 30 mm animal bone. There was a pelvic abscess with local fecal contamination but no generalized peritoneal soiling. An intraoperative inotrope requirement and the gross fecal peritoneal soiling mandated the performance of Hartmann’s procedure and the removal of the extruding bone.

The patient made an excellent recovery and was discharged from hospital. A histological assessment of the resected sigmoid colon demonstrated diverticulitis without evidence of malignancy. He will undergo elective reversal of his Hartmann’s procedure after colonoscopy.

## Discussion

Colonic diverticulosis is a disease affecting half the population over the age of 60 years [4]. The colonic outpouches are "pseudodiverticula" because not all layers of the bowel are involved. Mesenteric blood vessels create focal points of weakness as they pass through the layers of the bowel wall. The development of diverticulosis is thought to be related to excessively high intra-luminal pressures as a result of reduced fiber intake [5-6]. Active inflammation secondary to the perforation of a diverticulum or multiple diverticula is termed acute diverticulitis [4]. Contemporary management of acute presentations is determined according to disease severity. Uncomplicated episodes usually resolve without invasive treatment, whereas complicated disease (abscess formation or disseminated peritoneal soiling) often requires invasive treatment. The management of the latter group can be guided by the Hinchey classification ranging from percutaneous drainage to operative intervention [7].

A recent systematic review reported 52 published cases of foreign bodies complicating diverticular disease [3]. This was most frequently manifested as diverticulitis (42%) followed by bowel perforation (33%). There are seven previous case reports of a chicken bone causing diverticular perforation [8-14]. Only three of the former cases were diagnosed by computed tomography (CT) before operation [11,13-14]. All patients with chicken bone-induced diverticular perforation were treated surgically. The majority underwent laparotomy with some form of colonic resection whilst one patient had a laparoscopic washout and one had laparotomy with primary repair of the sigmoid colon defect [8,13]. One patient was managed with a subtotal colectomy and ileorectal anastomosis [9], two patients had a sigmoid colectomy [12,14], and two patients received Hartmann’s procedure [10-11]. There was one death from multiorgan dysfunction after Hartmann’s procedure although it is noted that this patient was an octogenarian with concomitant liver cirrhosis [11].

In a patient presenting with hypogastric abdominal pain, a complication of diverticular disease is high on the differential diagnosis list. The symptoms are likely to lead to cross-sectional imaging, which usually confirms the diagnosis. It has been suggested that colonic diverticulosis may predispose to foreign body entrapment because of its associations with strictures, pockets, and increased intra-luminal pressure [3]. In the rare event that the clinical picture is caused by a perforating chicken bone, this should be detected on imaging. The majority of reported cases were diagnosed at laparotomy but only one of these was published within the last 10 years. As CT imaging has become more widely available, one would expect that future cases of this condition would be initially detected by radiological means. Patient reporting of foreign body ingestion is not necessarily a useful test for determining sigmoid diverticular perforation. Unfortunately, patients do not always recall eating the offending object, as was the case with our patient [13].

There are cases of patients with intra-luminal animal bones and uncomplicated diverticulitis being managed successfully with endoscopic foreign body retrieval [3]. Given the patient presented in this report was clinically unstable, he received prompt surgical management by way of Hartmann’s procedure. It is recommended that all cases of bowel perforation be managed operatively. In the stable patient, initial laparoscopy for removal of the foreign body and assessment of peritoneal contamination is a reasonable approach. This was employed by one group of surgeons who staged the involved abdomen as Hinchey grade II and completed a washout without colon resection as per current practice [13].

## Conclusions

The diagnosis of diverticular perforation by an ingested foreign body is difficult to make clinically, given its tendency to mimic diverticulitis and other common diseases. The use of cross-sectional imaging is likely to detect the presence of an animal bone within the sigmoid colon. Whilst the majority of ingested foreign bodies pass eventually, entrapped or symptomatic foreign bodies may require intervention. The diagnosis of a foreign body and diverticulitis should concern clinicians, as these patients are likely to experience a progressive and complicated course that may require operative intervention. The management of this is much the same as for complicated diverticulitis, with the important difference that the removal of any foreign body should be of paramount concern. Other aims of treatment include the assessment of contamination and perforation and the resection of the affected colon if required as per the accepted principles used in the Hinchey classification system.

## References

[1] Morris AM, Regenbogen SE, Hardiman KM, Hendren S (2014). Sigmoid diverticulitis. JAMA.

[2] Shahedi K, Fuller G, Bolus R (2013). Long-term risk of acute diverticulitis among patients with incidental diverticulosis found during colonoscopy. Clin Gastroenterol Hepatol.

[3] Ross E, McKenna P, Anderson J (2017). Foreign bodies in sigmoid colon diverticulosis. Clin J Gastroenterol.

[4] Feuerstein JD, Falchuk KR (2016). Diverticulosis and diverticulitis. Mayo Clinic Proc.

[5] Camilleri M, Lee JS, Viramontes B, Bharucha AE, Tangalos EG (2000). Insights into the pathophysiology and mechanisms of constipation, irritable bowel syndrome, and diverticulosis in older people. J Am Geriatr Soc.

[6] West B (2006). The pathology of diverticulosis: classical concepts and mucosal changes in diverticula. Clin J Gastroenterol.

[7] Hinchey E, Schaal P, Richards G (1978). Treatment of perforated diverticular disease of the colon. Adv Surg.

[8] Akhtar S, McElvanna N, Gardiner KR, Irwin ST (2007). Bowel perforation caused by swallowed chicken bones- a case series. Ulster Med J.

[9] Glasson R, Haghighi KS, Richardson G (2002). Chicken bone perforation of a sigmoid diverticulum. ANZ J Surg.

[10] Gomez N, Roldos F, Andrade R (1997). Intestinal perforation caused by chicken bone mimicking perforated colonic diverticulitis [Article in Spanish]. Acta Gastroenterol Latinoam.

[11] Kornprat P, Langner C, Mohadjer D, Mischinger H (2009). Chicken-bone perforation of a sigmoid colon diverticulum into the right groin and subsequent phlegmonous inflammation of the abdominal wall. Wien Klin Wochenschr.

[12] Mapelli P, Head L, Conner W, Ferrante W, Ray J (1980). Perforation of colon by ingested chicken bone diagnosed by colonoscope. Gastrointest Endosc.

[13] Owen H, Nisaharan S, Amey A, Katherine D, David M (2010). Laparoscopic management of foreign body perforation in diverticular disease. Ann R Coll Surg Engl.

[14] Rasheed A, Deshpande V, Slanetz P (2001). Colonic perforation by ingested chicken bone. AJR Am J Roentgenol.

